# Preschool Children’s Processing of Events during Verb Learning: Is the Focus on People (Faces) or Their Actions (Hands)?

**DOI:** 10.3390/brainsci12030344

**Published:** 2022-03-03

**Authors:** Jane B. Childers, Emily Warkentin, Blaire M. Porter, Marissa Young, Sneh Lalani, Akila Gopalkrishnan

**Affiliations:** Department of Psychology, Trinity University, San Antonio, TX 78212, USA; ewarkent@trinity.edu (E.W.); blairemporter@utexas.edu (B.M.P.); myoung5@trinity.edu (M.Y.); sneh.lalani@gmail.com (S.L.); akila.gopalkrishnan@gmail.com (A.G.)

**Keywords:** verbs, eye tracking, word learning, comparison

## Abstract

Verbs are central to the syntactic structure of sentences, and, thus, important for learning one’s native language. This study examined how children visually inspect events as they hear, and do not hear, a new verb. Specifically, there is evidence that children may focus on the agent of the action or may prioritize attention to the action being performed; to date, little evidence is available. This study used an eye tracker to track 2-, 3-, and 4-year-olds’ looking to the agent (i.e., face) vs. action (i.e., hands) while viewing events linked to a new verb as well as distractor events. A Tobii X30 eye tracker recorded children’s fixations to AOIs (head/face and hands) as they watched three target events and two distractor events in different orders during the learning phase, and pointed to one of two events in two test trials. This was repeated for a second novel verb. Pointing results show that children in all age groups were able to learn and extend the new verbs to new events at test. Additionally, across age groups, when viewing target events, children increased their looking to the hands (where the action is taking place) as those trials progressed and decreased their looking to the agents’ face, which is less informative for learning a new verb’s meaning. In contrast, when viewing distractor events, children decreased their looking to hands over trials and maintained their attention to the face. In summary, children’s visual attention to agents’ faces and hands differed depending on whether the events cooccurred with the new verb. These results are important as this is the first study to show this pattern of visual attention during verb learning, and, thus, these results help reveal underlying attentional strategies children may use when learning verbs.

## 1. Introduction

Verbs are central to the syntactic structure of sentences. A controversy in this area is whether children focus on the agent of the action or the action being performed, and to what extent they focus on each one. Some studies suggest that young children learn verbs best when a single agent is seen, which could mean that they are attending too much to agents rather than the actions while learning verbs [1,2]. In other studies, children seem to be attending to actions [3] or results, e.g., [4,5]. Additionally, in everyday contexts, children often see events linked to a new verb that are interspersed with distracting events. Yet most laboratory studies of verb learning show children relevant events as they hear the new verb. The present study is important because few studies have tracked children’s looking patterns during verb learning, and few studies have included distracting interleaved events. By tracking looking to target and distractor events, these results will reveal whether children adjust their visual attention to agents (faces) or their actions (hands) differently depending on whether the event is linked to a new verb, which is important for understanding how they may be processing events during verb learning.

To accurately acquire a verb, learners must solve what Gleitman and Gleitman [6] described as the “packaging problem”, wherein learners must package together only the relevant aspects of a dynamic event and disregard any irrelevant information. Given the difficulty of this problem, learners often use information across events and sentences or engage in cross-situational learning, e.g., [7,8,9,10]. Research has shown that the comparison of events can help children learn and generalize verbs, e.g., [3,11,12,13,14]. Additionally, children can benefit from seeing similar or varied events, depending on the nature of the task and test conditions [5,11,12,15,16]. In light of this prior research, it seems clear that young children can glean information across a set of events as they learn verbs, but theories differ in the mechanisms they posit that underlie this cross-situational processing.

Two key theories in this area are statistical learning and structural alignment. In statistical learning, observers compare events by noting co-occurrences between specific words and referents, e.g., [17,18,19]. In structural alignment, observers compare events linked to a new verb over instances by aligning elements across the instances based on their common relational structure, e.g., [20,21,22]. Our study tests whether children’s looking at target and distracting events during the learning phase differs in terms of their focus on agents (faces) or actions (hands). By including both types of trials, we can ask whether there are general looking preferences (e.g., for faces or hands) or whether children attend to events differently when viewing events linked to verbs vs. distracting events. To our knowledge, no prior study verb has used eye tracking in this way, though Scott and Fisher, in 2012 [3], showed that 2 ½-year-olds could track which whole event was linked to a specific verb over trials.

The present research is related to a recent set of studies in which we showed 2- to 4-year-old children the same events as those used in the present study (without an eye tracker) [23]. Children were assigned to different orders of events. In all orders, they saw five events during a learning phase; three were target events and two were distractor events. When tested in a forced choice task, children as young as 2 ½ years were able to extend the new verb, demonstrating an ability to distinguish between target and distractor events. However, without an eye tracker, we only have indirect evidence of children’s ability to ignore distracting events. Although this eye tracking study builds on a prior study conducted with an iPad [23], that prior study used complex, naturalistic backgrounds in the video stimuli, including a park and a kitchen scene. To reduce the visual clutter for the eye tracking study, we re-filmed the events against a plain colored wall. Additionally, the iPad screen used in the prior study was smaller than the large monitor linked to the eye tracker used for this study.

We used a Tobii x30 eye tracker to track children’s looking during the learning phase to the agents’ head/face vs. their hands within each event. As no prior study has tracked looking to the face vs. hands, we hypothesized that children would look more to the hands as the hands AOI (area of interest) is larger (in our stimuli and in everyday life) than the face AOI, and the hands are moving. However, two prior studies demonstrate that children can also be overly attentive to the agent in an event, performing fewer verb extensions in events with multiple agents [2], particularly when events were more complex [1]. Given that these two studies suggest a cost from attending to agents, it could be that children will attend more to the face/head of an agent than to the hands during verb learning. Attending more to the hands would help in verb learning while attending to the face would not.

Thus, in the study we used the eye tracker to ask whether children focused more on the face/head or the hands while seeing events in the learning phase, and whether this differed depending on whether the event was linked to a new verb (target) or was not (distractor). We also asked whether they could extend the new verbs at test.

## 2. Participants

Twenty-four 2 ½-year-old children (Mage = 2;8; range: 2;0–2;11; shortened to 2-year-olds for clarity), thirty-one 3 ½-year-old children (Mage = 3;4; range: 3;0–3;11) and twenty-one 4 ½-year-old children (Mage = 4;4; range: 4;0–4;10) participated in this study with 40 girls and 36 boys. Most children were from middle income or upper-middle income homes. Of the families who provided race or ethnicity information, 25 reported their ethnicity as White, eight reported their ethnicity as Hispanic, seven chose White and Hispanic, one participant reported their ethnicity as Asian, and one chose Asian and Hispanic. Children were excluded if their parents reported their exposure to English was less than 80% and if their teachers reported a speech delay. All children had normal vision or corrected vision. Additional children participated but were excluded from the final sample because there was an equipment failure either in the presentation of the stimuli or in capturing the eye tracking data (17) or the child got out of the chair during the study (1). This study’s procedures were approved by the Trinity University Institutional Review Board.

## 3. Materials

**Figure 1:**
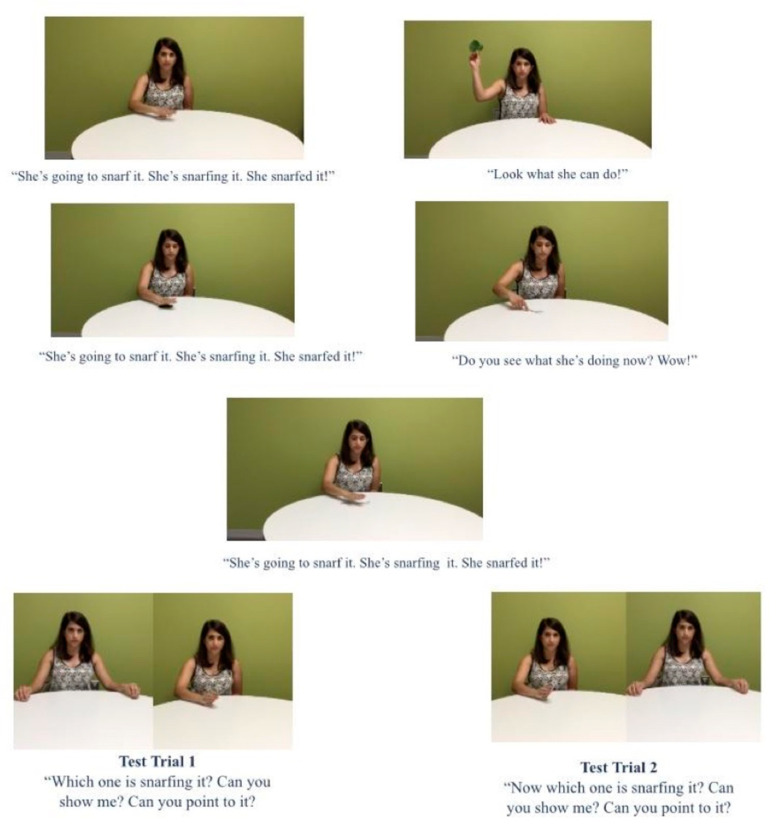
Example Stimuli. The top five photos show five events children saw during the learning phase in the study: Target (**upper left**), Distractor (**upper right**), Target (**middle left**), Distractor (**middle right**), and Target (**middle center**). The bottom two pairs of events show test trials with Test trial 1 (**left; correct extension right photo**) and Test trial 2 (**right; correct extension left photo**).

Video stimuli were created showing female actors performing three target scenes showing a single causative action and two distinct distractor actions for each of two novel verbs (see Figure 1). For the test scenes, a new target scene and a new distractor scene were also filmed.

Specifically, two sets of videos were filmed in which actors used their hands/bodies or tools to enact events that could be done in a park (see Appendix A). For example, in one set (Park 1), an actor picks up a natural object so that it sticks to an open hand (picking up a leaf, a stick, and a rock in the target events), and waves a leaf around and twirls a stick on the table using her finger in the two distractor events. At test, children saw her pick up an object using an open hand (correct) or move an object from the center to the sides of the table (incorrect). A second event set from this park setting (Park 2) showed the novel target event of sifting objects using a porous flat object to sift sand (target events showed different porous objects and included rocks instead of sand); distractor events showed the agent throwing a rock into the air and tapping two sticks together. At test, children saw the agent wave a sticky note in the air (incorrect) or sift a new object (correct).

There were also two events from a kitchen context. In one set, an actor uses her finger to trace a pattern into oatmeal, into chocolate frosting, and into rice in the three target events (Kitchen 1), and she scoops up oatmeal with her hands and wipes chocolate off her finger onto the side of a container in the two distractor events. At test, she is seen using her finger to trace a pattern into ketchup (correct) vs. holding a ketchup bottle and squirting ketchup into the tray (incorrect). A second kitchen-related set of events (Kitchen 2) was also filmed with the same structure (target event, dunking different objects into a bowl of liquid using different tools); distractor events showed the agent removing a paper towel from a roll and dipping a towel into an empty container. At test, children saw a new dipping action (correct) and saw the agent crushing a cracker using tongs (incorrect).

A three second black screen was inserted between each of the events both during the learning and test phases. A script was created so that for each scene, an experimenter would direct attention to the target actions by saying “Look! She’s going to <novel verb> it. She’s <novel verb>-ing it! She <novel verb>-ed it!”, while using non-labeling speech for the distractor events (e.g., “Oh, look what she is doing!”).

**Figure 2:**
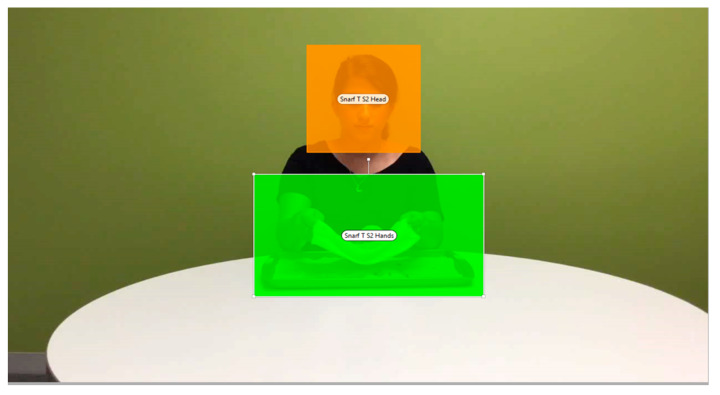
Example of AOIs drawn for the head region and the hands region.

Areas of Interest (AOIs) were drawn by hand using Tobii Studio, with separate AOIs drawn around the head/face region and the hands region for each scene (see Figure 2).

In addition, because we initially conducted a study using similar stimuli (with cluttered backgrounds) on an iPad, when we reviewed the scenes that were distractor scenes and target scenes for each verb for this eye tracking study, we found that the scenes differed in length. As we wanted to compare distractor to target scenes, we manually adjusted the activation window for each scene to be equivalent across the two types of scenes. Although it would have been better to edit the scenes to be exactly the same length, by adjusting the activation window in the Tobii Studio software for each event, we ensured that the tracker recorded children’s looking for the same amount of time in the distractor and target scenes.

## 4. Design

Sets of events were constructed so that each participant could learn two verbs: one from each context (one from the two kitchen events, K1 or K2, and one from the two park events, P1 or P2, see Appendix A). Different children saw different sets to minimize the influence of a single set of events on the results. We also created three orders of the events: Target first (TTDDT), Distractor first (DTTDT), and Alternating (TDTDT) where T represents a Target event and D represents a Distractor event (see Figure 1); children were assigned randomly to one of these three orders. These orders represent all the possible orders of the five events, keeping the last two events constant.

## 5. Procedure

After building rapport with the children at their childcare center, children whose parents had returned a signed consent form were taken to a quiet room. Children sat in front of a 21-inch flat screen video monitor. A Tobii X30 eye tracker device was placed on the bottom edge of the monitor and was connected to a laptop. Attached to the top of the monitor was a webcam that recorded the children’s pointing responses for later coding. The distance between the table holding the video screen and tracker and the participant’s chair was approximately 16 inches, with some variation to maximize an individual participant’s calibration. The eye tracker used a corneal reflectance tracking technique to measure eye movements. A near infrared light source was directed at the participant, undetectable to the naked eye, and the reflection of the light on the cornea was recorded as the participant watched the video stimulus on a monitor. After being seated in front of the video monitor, the experimenter calibrated the Tobii X30 eye-tracker using the Tobii 5 point calibration stimuli for infants; the software used throughout was Tobii Studio.

Two experimenters were present: one controlled the eye tracker using a laptop and used a script to produce the stimulus sentences, and the other coded children’s pointing using a score sheet. A recording of the session was captured using a webcam mounted above the 21-inch flat screen video monitor for later coding of the pointing responses from video.

Participants were first shown two warm-up trials in which they were asked to point to a familiar event out of two possible events. In one warm-up, children were asked to point to one of two static objects: while seeing a bunch of grapes (left) and a toy teapot (right) they were asked to point to the grapes (“Can you point to the grapes?”). The pair was shown again with the side of the correct match reversed and the question was repeated (“Now look. Where are the grapes now?”). They were then asked to point to one of two dynamic events: a hand pouring pretend liquid into a cup (left) vs. a hand making a cow walk (right); they heard “Can you point to the cow?” and “Where’s the cow now?”.

## 6. Learning Phase

Each child was then shown two sets of events, one at a time. They heard two novel verbs: *gorp* and *snarf* (one verb for each set). In each set, children saw three relevant events (T) and two distractor events (D) in one of three orders while hearing the experimenter produce the new verbs using a script. During the relevant events, they heard the novel verb three times (“She is going to ___ it. She is ___ing it. She ___ed it.”) while during the distractor events, they heard non-labelling speech (“Oh look what she’s doing.”).

## 7. Test Phase

At test, children were shown a split screen with two different events while hearing “Now it’s your turn to find gorping” (or “snarfing” in the other set). Participants were then asked to point to which verb was correct, hearing “Point to <verb>ing. Can you point to the one who’s <verb>ing?” (see Figure 1). In the second test trial, the same two videos were shown on the opposite sides of the screen and children heard “You get one more turn to play the game. Can you point to <verb>ing? Which one is <verb>ing?”) Thus, two test trials were shown for each verb and the correct side of the screen was counterbalanced so that the correct answer appeared equally as often on the left and right side of the screen. If children did not respond, the video was paused to give them more time to respond. This process was repeated for a second verb.

## 8. Coding

An initial coding of the pointing responses was collected during the experimental session. Second and third codings were then conducted from video; all three coders were independent. If the participant did not respond, their response was left blank and not coded as incorrect. This allowed us to analyze whether children were correct on the trials in which they responded. Interrater reliability calculated between the second coder and a third coder, both from video, showed 94% agreement with Cohen’s kappa = 0.88 (almost perfect agreement [24]).

## 9. Results

### 9.1. Pointing Results

A univariate ANOVA with Age group (3: 2 s, 3 s, 4 s) and Order (3: Target first, Distractor first, Alternating) as between subjects factors, dv = proportion trials correct (number correct/total trials with responses), showed that there was a main effect of Age group, *F* (2, 75) = 3.94, *p* = 0.024, η^2^ = 0.11, and an Age group by Order interaction, *F* (4, 75) = 3.04, *p* = 0.023, η^2^ = 0.15. Given the significant Age group by Order interaction, we split the data by age group and repeated the univariate ANOVA within each age group, following up with one sample *t*-tests to compare responses to chance (=0.50 correct).

In these univariate ANOVAs within each age group, no significant effects of Order emerged in the 2-year-olds and 4-year-olds data. There was a significant effect of Order in the 3-year-old group, with Order, *F* (2, 30) = 4.25, *p* < 0.05, η^2^ = 0.23; pairwise comparisons with Sidak corrections show that children in the Distractor first (DTTDT) condition were significantly less successful at test than were children in the Alternating condition (TDTDT). As this result was found only in one of the three age groups, it suggests to us that overall, the order of the events did not exert a major effect on children’s pointing responses.

**Figure 3:**
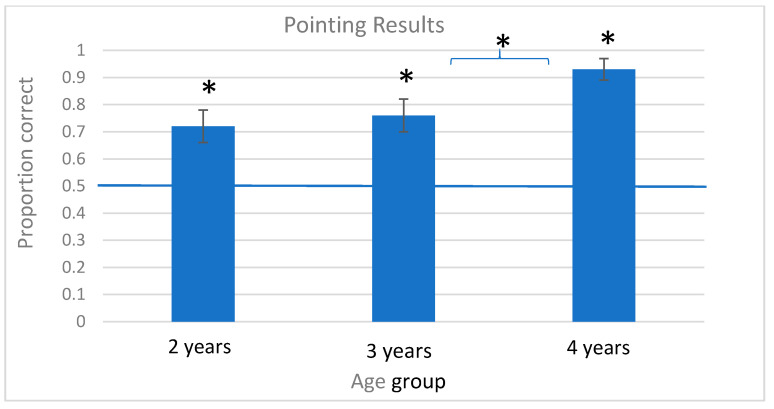
Pointing results, * *p* < 0.05, error bars show SEM, blue line represents chance responding.

A separate analysis examined whether children’s pointing responses differed from chance. One sample *t*-test showed that children in all three age groups exceeded chance. (All *t*-tests reported are two-tailed.) Specifically, 2-year-old children’s responses exceeded chance (*M*prop = 0.72, SE = 0.06), *t*(23) = 3.40, *p* = 0.002, as did 3-year-old children’s (*M*prop = 0.76, SE = 0.06), *t*(30) = 4.60, *p <* 0.001, and 4-year-old children’s responses (*M*prop = 0.93, SE = 0.04), *t*(20) = 12.21, *p* < 0.001. Reflecting on this pattern of responses, the 2- and 3-year-olds’ pointing responses are similar to each other, but 4-year-olds appeared to be responding at a significantly higher rate. Indeed, an independent samples *t*-test comparing 3-year-olds’ to 4-year-olds’ responses was significant, *t*(50) = −2.25, *p* = 0.029 (see Figure 3).

Overall, the pointing results show that children in all age groups learned and extended the new verbs successfully, order of learning events was only important in the 3-year-old age group, and the main developmental change occurred between 3 and 4 years.

### 9.2. Eye Tracking Results: Looking to the Face vs. the Hands

Before analyzing the looking data, we reviewed the quality of the eye tracking obtained and excluded additional participants which the tracker did not track successfully at least 30% of the time (*n* = 6). Four participants’ eye movements could be tracked but did not point and are only included in the following eye tracking analyses.

A repeated measures analysis of variance (ANOVA) was computed with Age group (3: 2, 3, 4 years) as the between-subjects factor and Trial type (2: target, distractor), Trial number (2: first, last), and AOI (2: face, hands) as within-subjects factors; dv = total fixation duration (with zeros). This analysis revealed a significant main effect of Trial type, *F* (1, 67) = 18.78 *p* < 0.001, η*_p_*^2^ = 0.22, Trial number, *F* (1, 67) = 12.96, *p* < 0.001, η*_p_*= 0.16, and AOI, *F* (1, 67) = 229.29, *p* < 0.001, η*_p_* ^2^ = 0.77; there was no significant main effect of Age. There were also three two-way interactions: Trial type × Age group, *F* (2, 67) = 4.66, *p* = 0.013, η*_p_*^2^ = 0.12, Trial type × Trial number, *F* (1, 67) = 10.47, *p* = 0.002, η*_p_*^2^ = 0.14, and Trial type × AOI, *F* (1, 67) = 135.49, *p* < 0.001, η*_p_*^2^ = 0.67. Finally, there was a three-way interaction of Trial type × Trial number × AOI, *F* (1, 67) = 27.36, *p* < 0.001, η*_p_*^2^ = 0.29. To analyze the three-way interaction, we split the data by Trial type and re-ran the analyses.

**Figure 4:**
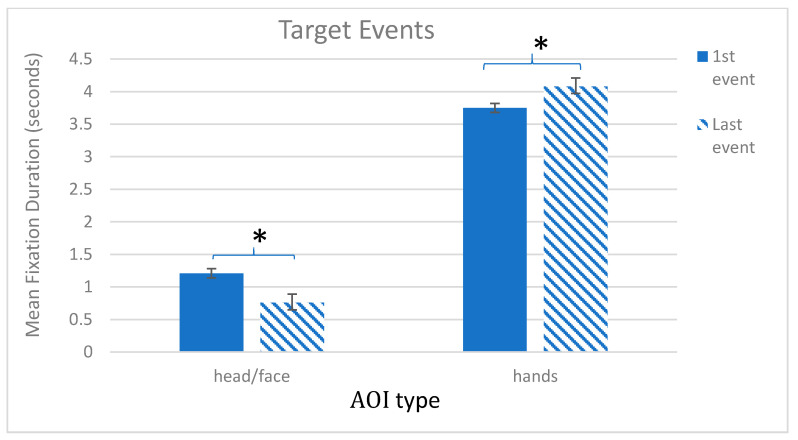
Target Events. Graph shows mean total fixation duration by trial (first, last) and AOI type (head/face, hands), * *p* < 0.05.

A repeated measures ANOVA examining looking during the Target trials revealed no effect of Trial number, *F* (1, 69) = 0.91, *ns*, but a main effect of AOI, *F* (1, 69) = 580.62, *p* < 0.001, η*_p_*^2^ = 0.89, and a significant Trial number × AOI interaction, *F* (1, 69) = 30.14, *p* < 0.001, η*_p_*^2^ = 0.30. Pairwise comparisons with Sidak corrections showed that, in both the first and last target events, children looked longer at the hands than the face. Specifically, in the first Target event, looking to hands (*M* = 3.75, SE = 0.13) was greater than face (*M* = 1.21, SE = 0.07), *p* = 0.003 (2 sided), and in the last target event, looking to hands (*M* = 4.08, SE = 0.11) was greater than face (*M* = 0.76, SE = 0.07), *p* < 0.001. Importantly though, across trials, looking to the hands increased, *p* = 0.003, while looking to the face significantly decreased, *p* < 0.001 (see Figure 4).

**Figure 5:**
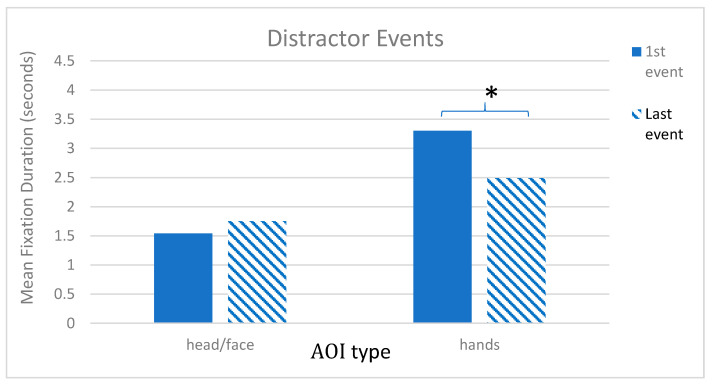
Distractor Events. Graph shows mean total fixation duration by Trial (first, last) and AOI type (head/face, hands), * *p* < 0.05.

In the distractor events, a different pattern emerged. A repeated measures ANOVA revealed a main effect of trial number, *F* (1, 69) = 26.96, *p* < 0.001, η*_p_*^2^ = 0.28, a main effect of AOI, *F* (1, 69) = 53.91, *p* < 0.001, η*_p_*^2^ = 0.44, and a trial number × AOI interaction, *F* (1, 69) = 14.10, *p* < 0.001, η*_p_*^2^ = 0.17. Pairwise comparisons with Sidak corrections showed that, as in the target trials, during these distractor events, children looked longer at the hands than the face with hands (*M* = 3.30; SE = 0.13) greater than face (*M* = 1.54; SE = 1.0) in the first distractor event, *p* < 0.001 (2 sided), and hands (*M* = 2.49; SE = 0.15) greater than face (*M* = 1.75; SE = 0.14), *p* = 0.004 (2 sided) in the second distractor trials. However, across trials, a different pattern was seen with children’s looking to the hands decreasing, *p* < 0.001, while looking to the face was maintained (see Figure 5).

## 10. Discussion

These results are the first to show that children visually attend to events that are linked to a new verb differently than they attend to distracting events. This is important because children learning verbs often see other intervening events as they are learning verbs (e.g., seeing a stirring event while learning the verb ‘chop’ in the kitchen), which need to be processed differently than events linked to the target verb. Specifically, across age groups, when viewing target events, children increase their looking to the hand region over trials (where the action is taking place) and decrease their looking to the agents’ face, which is less informative for learning a new verb’s meaning. In contrast, when viewing distracting events, children decrease their looking to hands over trials (i.e., the action) while maintaining their attention to the head/face region. Thus, in the distractor events, they are looking less at what the agent is doing as they see more trials, which should help them ignore those events as they learn a verb, whereas when seeing relevant events, they are increasing their looking to the agent’s actions, which should be helpful for deducing what a new verb means.

These results add to a body of research showing that children can compare events during verb learning, and that comparisons help them extend new verbs, e.g., [3,14,15]. What these results add is evidence that children are adjusting their visual fixations differently as they see events linked to a new verb as opposed to distracting events, suggesting that they are strategic in how they visually inspect events. These new results also add to the few studies that have used eye tracking to study children’s verb learning. They extend the results of Papafragou et al., 2021 [25], by including novel verbs and focusing on faces and hands, adding to their evidence of children’s attention to manners and paths. They add to the findings of Valleau et al., 2018 [26], by including tracking to parts of events vs. whole events, and they add to a study from Childers et al., 2016 [15], that included different types of events. That study tested whether children benefited from seeing similar than varied events as opposed to all varied events and showed that children seeing those similar events first increased their looking to important elements in events (e.g., agents, affected objects), and by age 3, could succeed in extending the new verbs at test only in this similar first condition. They also add to the Childers et al. 2020 [27] study which showed that children increased their visual attention to object types that varied across a learning set (tools and affected objects); this suggests children noticed which element varied likely because they were comparing elements across the events. Together, these studies [15,27] with the present result—showing greater attention to hands, and less attention to faces, only for relevant events—all show different ways children attend to specific elements of events as they learn verbs, which is important.

Specifically, because these results show differing attention to different elements in events, they seem more consistent with the structural alignment view which posits learners attend to individual elements and align those elements across instances, e.g., [21]. Although statistical learning could also include elements (as it has been shown to work for syllables [28]), to our knowledge, there is no current empirical evidence for attention to elements within events, or overt description of how children could compute statistics across elements within events during verb learning, within the statistical learning framework.

In terms of pointing responses at test, we found that across ages, children were able to learn these new verbs, pointing successfully to a new event (and not a distractor event) that fit the verb they heard during the learning phase at test. Our pointing results also show an increase in the ability to learn these verbs between the ages of 3 and 4 years. When we compare these results with our prior study with similar events containing cluttered, naturalistic backgrounds shown on a tablet, in both studies, children were able to learn and extend new verbs at test, even though the learning phase included two irrelevant events. In the prior study [23], 2-year-olds’ responses exceeded chance but also were significantly fewer than 3-year-olds’ responses; in the present study, these age groups performed similarly. This could suggest that 2-year-olds were distracted by the cluttered backgrounds in the earlier study, performing better without those backgrounds in this study. Furthermore, in the prior study, children succeeded in all three orders of events, and this same result was also (largely) found in this study. Thus, the results across the two mediums of stimulus presentation, iPad and eye tracker, converged and the pointing results were replicated. One difference in design was that the prior study included two control conditions, a salience control (test trials only), and a one event condition (one target event than test), and children seeing the comparison events were more successful in extending verbs than were children in the control conditions. As we had already demonstrated these differences, we did not include those control conditions in the present study.

Linking the present study to some basic findings concerning brain development (e.g., [29]), there is a brain area for processing face-like stimuli (the fusiform face area) which appears to help infants as young as 4 months old to process faces, and which appears to continue to develop during childhood. Given these general facts, it seems likely that this FFA guided our preschool children’s attention to the agent’s head/face during our study. In terms of attention to the hands, a similar area which is close to the FFA, the EBA (extrastriate body area), seems implicated in the visual processing of the human body and can take years to fully develop. Given this pattern of development, our preschoolers likely used the EBA to process the hand movements seen in our events, but may not have had adult-like processing in the EBA given its later development (up to 9–12 years).

Turning to limitations of our study, one limitation is that the agent stayed the same across trials. Thus, children could shift their focus from the face to the hands region as they saw more trials. That they did shift to looking more to the hands during the target events suggests that they recognized that the agent stayed the same. At the same time, when viewing the distractor events, children maintained their attention to the agent (i.e., her face/head) over trials (and decreased their attention to the hands), which is interesting. Future eye tracking studies should vary agents to show what effect varying agents have on children’s visual attention. Given the present results, we predict that children may maintain attention to the agent’s face in both target and distractor events if the agent varies, and, thus, may not be able to focus as efficiently on the agent’s hands in the target events as was seen in these results. This would support prior findings showing a cost for variability of agents in verb learning [1,2], and extend them by providing eye tracking evidence for this predicted attention cost.

Another limitation is that in this study, there were multiple cues to whether a scene was relevant or irrelevant for learning a verb. Distractor events were heard with non-labelling speech and depicted actions that differed markedly from relevant events. Therefore, future studies will need to separate these factors in order to understand which factor, or a combination of both, contribute to the participants’ understanding of the event as irrelevant. However, artificially presenting a verb during an irrelevant event or showing sets of irrelevant events that are highly similar to the events linked to the verb being learned could have made this task more confusing, especially for our youngest participants. We also do not know if these results extend beyond learning English, but these strategies for looking would be useful for learning verbs in any language, and, thus, we predict that they should.

## 11. Conclusions

In conclusion, learning new verbs is important to learning one’s native language. Our study suggests that by 2 ½ years old, children have developed visual strategies for inspecting events that should help them attend to events appropriately when seeing relevant events and hearing verbs (attending more to what the hands are doing than the face) and help them ignore distracting information (as they focus on faces and not hands) when they see distracting events. These are exciting new findings that reveal what mental mechanisms could underlie early verb learning. They also suggest ways to help children who may be experiencing a language delay and, perhaps, are not adjusting their visual attention in helpful ways. By directing their attention, or teaching them strategies for comparing events and ignoring events, therapists may be able to help children learn how to acquire new verbs.

Funding for this research was provided by a grant from the National Institute of Health (2R15 HD044447), the Murchison Scholars Fund, and the support of Trinity University. The project was supported by the Eunice Kennedy Shriver National Institute of Child Health and Human Development, and the content is solely the responsibility of the authors and does not necessarily represent the views of NICHD or NIH. We thank Victoria Bell, Sophia Arriazola, Aidan Burke, Priscilla Tovar-Perez, Hannah Bortz, Katy Capps, and Sophia Rodriguez for their assistance in data collection and coding.

## Data Availability

Data available from the first author upon request.

## Appendix A

**Table A1:** Target Event: “Gorp”.

**Set 1 (Kitchen 1: To Make a Pattern in a Gooey Substance)**		
**Target Events**	**Distractor Events**	**Test**
Agent dragged their finger through a pan full of sand	Sand was picked up from a pan full of sand and dropped	Agent dragged their finger through a pan full of ketchup (Correct)
Agent dragged their finger through a pan full of chocolate	Agent wiped their chocolate-covered finger on the edge of a pan full of chocolate	Agent squeezed a bottle of ketchup into a pan (Incorrect)
Agent dragged their finger through a pan full of oats		
**Set 2 (Kitchen 2: To Dunk Something into a Liquid Using a Tool)**		
**Target Events**	**Distractor Events**	**Test**
Agent dipped a paper towel into a bowl of green liquid with a bag clip	Agent pulled one paper towel sheet from the paper towel roll with a bag clip	Agent dipped a square cracker into a blue bowl of green liquid with a bag clip (Correct)
Agent dipped a square piece of paper into a bowl of green liquid with a bag clip	Agent picked up a square piece of paper	Agent rushed a square cracker with a bag clip (Incorrect)
Agent dipped a piece of bread into a bowl of green liquid with a bag clip		

**Table A2:** Target Event: “Snarf”.

**Set 1 (Park 1: To Pick Up Something Using an Open Hand)**		
**Target Events**	**Distractor Events**	**Test**
Agent picked up a pebble using the palm of their hand	A stick was pushed in a circle with one finger	Agent picked up a rock using an open hand (Correct)
Agent picked up a stick using the palm of their hand	A leaf was picked up and waved in the air	Two rocks in the center of the table were picked up and placed on two opposite sides of the table (Incorrect)
Agent picked up a leaf using the palm of their hand		
**Set 2 (Park 2: To Sift Objects Using a Tool)**		
**Target Events**	**Distractor Events**	**Test**
Agent used a paper towel to sift two large rocks over a pan full of sand	Agent picked up a rock placed on a pan and tossed it in the air twice	Agent sifted sand and leaves over a pan full of sand (Correct)
Agent sifted sand and grass over a pan with sand	Agent hit two sticks together in an X-shape over a pan with sand and sticks	Agent waved a leaf over a pan with sand (Incorrect)
Agent sifted two small rocks using a paper towel over a pan full of sand		
